# UEFA Euro 2020: lessons from the first multi-city international mass gathering during the COVID-19 pandemic

**DOI:** 10.1017/S095026882200156X

**Published:** 2022-11-17

**Authors:** Kazim Beebeejaun, Richard Pebody, Silviu Ciobanu, Jukka Pukkila, Catherine Smallwood, Ihor Perehinets

**Affiliations:** 1 Health Emergency Information and Risk Assessment, World Health Organization Regional Office for Europe, Copenhagen, Denmark; 2 World Health Organization Health Emergencies Programme, World Health Organization Regional Office for Europe, Copenhagen, Denmark; 3 Country Health Emergency Preparedness and International Health Regulations, World Health Organization Regional Office for Europe, Copenhagen, Denmark

**Keywords:** COVID-19, outbreaks

## Abstract

Mass gatherings (MG) present a number of challenges to public health authorities and governments across the world with sporting events, tournaments, music festivals, religious gatherings and all other MG having historically posed a risk to the spread and amplification of a range of infectious diseases. Transmission of gastrointestinal, respiratory, waterborne and sexually transmitted infectious diseases pose a particular risk: all have been linked to MG events [1–4]. Infection risk often depends on the nature of the mass gathering, and on the profile and behaviour of its participants. The interaction between environmental, psychological, biological and social factors plays a vital part. The risk of outbreaks particularly as a result of respiratory transmission remains high at MG, with the majority of outbreaks over the last two decades resulting from a variety of respiratory and vaccine preventable pathogens [5–7]. Concerns about the spread of infectious diseases at MG are often focussed on crowding, lack of sanitation and the mixing of population groups from different places. Sporting events, which have in recent decades become more complex and international in nature, pose a challenge to the control of communicable disease transmission [8]. Despite this, large scale outbreaks at sporting events have been rare in recent decades, particularly since the rise of more robust public health planning, prevention, risk assessment and improved health infrastructures in host countries [9].

## Introduction

Mass gatherings (MG) present a number of challenges to public health authorities and governments across the world with sporting events, tournaments, music festivals, religious gatherings and all other MG having historically posed a risk to the spread and amplification of a range of infectious diseases. Transmission of gastrointestinal, respiratory, waterborne and sexually transmitted infectious diseases pose a particular risk: all have been linked to MG events [1–4]. Infection risk often depends on the nature of the mass gathering, and on the profile and behaviour of its participants. The interaction between environmental, psychological, biological and social factors plays a vital part. The risk of outbreaks particularly as a result of respiratory transmission remains high at MG, with the majority of outbreaks over the last two decades resulting from a variety of respiratory and vaccine preventable pathogens [5–7]. Concerns about the spread of infectious diseases at MG are often focussed on crowding, lack of sanitation and the mixing of population groups from different places. Sporting events, which have in recent decades become more complex and international in nature, pose a challenge to the control of communicable disease transmission [8]. Despite this, large scale outbreaks at sporting events have been rare in recent decades, particularly since the rise of more robust public health planning, prevention, risk assessment and improved health infrastructures in host countries [9].

During the coronavirus disease 2019 (COVID-19) pandemic, an unprecedented number of cancellations and postponements of MG occurred in order to mitigate the potential spread of severe acute respiratory syndrome coronavirus 2 (SARS-CoV-2) at such events. Some of the most well-known included the cancellation of the annual Hajj pilgrimage for the first time in over 160 years, the 2020 Tokyo Summer Olympics, the Six Nations Rugby championship and the Glastonbury music festival.

The Union of European Football Associations' (UEFA) European Football Championship is a major quadrennial football tournament that regularly draws millions of fans from across the world to attend. The 2016 tournament hosted by France (also known as Euro 2016) had an estimated 2.4 million fans attend matches and over 5 billion viewers around the world. Euro 2020, postponed by one year to June 2021 due to the pandemic, posed a number of unique challenges to public health planning, surveillance and response. First, the tournament which has been running since 1960 is typically hosted by a single country in Europe. However, as a celebration of the 60th anniversary of the tournament, organisers opted for the tournament to be hosted by 11 different countries across Europe. Second, as the tournament was one of the first major international MG with attendees to have taken place since the pandemic began, the different public health and social measures implemented, varied travel restrictions and requirements in place across countries, and the rapid changes in local incidence of COVID-19 posed challenges not only from a public health perspective but also logistically and politically. Despite these issues, with many countries in Europe in a phase of re-opening and travel restrictions changing on a weekly basis, the tournament represented one of the first opportunities for sporting MG to recommence and for a number of lessons to be learnt on how MG might influence COVID-19 epidemiology in different transmission and Public Health And Social Measures (PHSM) contexts.

## Challenges and risks associated with euro 2020

**Fig. 1.**
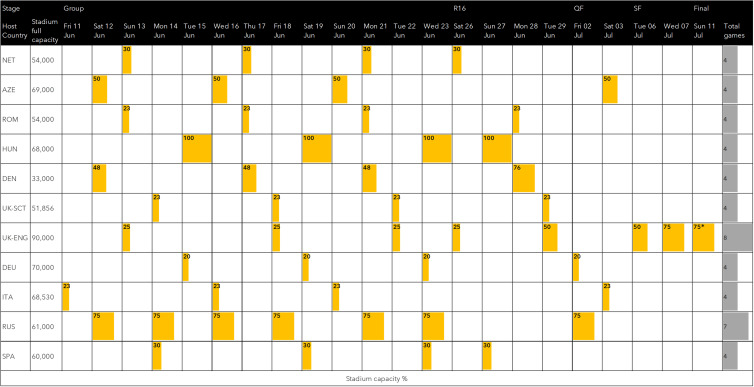
Stadium capacity restrictions during UEFA Euro 2020, 11 June – 11 July 2021. * Stadium capacity limits were unable to be enforced due to mass security incident.

The tournament consisted of 24 national teams playing 51 matches across 11 different host countries in the WHO European Region between 11 June and 11 July 2021 (Fig. 1). Each host had a unique set of public health risks associated with hosting matches including: (1) Incidence and trends of COVID-19 before and during the tournament; (2) Strictness of local PHSM and adherence levels; (3) Restrictions and mitigation measures inside stadia and (4) Public health surveillance capacity.

### COVID-19 incidence

In the month preceding the start of the tournament, incidence of COVID-19 was generally decreasing or stable in most countries, with 4/11 host countries (Hungary, Denmark, England and Scotland) having modelled increasing or likely increasing trajectories [10]. The highest 7-day incidence rates per 100 000 population of COVID-19 were reported by the following host cities: Glasgow (141), St. Petersburg (111), Copenhagen (109) and Seville (103). Lowest incidence rates were reported in Azerbaijan (6), Bucharest (7) and Budapest (9) [11].

### Mitigation measures

#### Inside stadia

Public health and social measures varied in levels of strictness and generally aligned with the host country's levels of restrictions at the time (Table 1). Measures inside stadia were determined and enforced by host organisations in partnership with UEFA – these included seating capacity limits, social distancing, negative test entry requirements and mask mandates.

**Table 1.** Public health and social measures inside stadia

Measure	Host requirements
Social distancing	Required by all hosts in communal areas of stadia.
Masks	Required by all hosts at all times inside stadia, both seated and unseated.
Pre-entry testing	The majority of hosts (8/11) required a negative PCR test or proof of vaccination prior to entry. Baku, Glasgow and Seville had no entry requirements.
Stadium capacity limits	Stadium capacities varied according to host country and stage of tournament. In the earlier group stages of the tournament (36 matches), all hosts except for Budapest opted for stadium capacities at or below 50% (33/36), with a median capacity of 30% observed (range: 23–100%) (Fig. 1). During the advanced stages of the tournament (15 matches), hosts opted for higher stadium capacities with 7/15 matches operating at or above 50% capacity with a median capacity of 48% (range 20–100%). The last 3 matches of the tournament in London operated at 75% capacity.

#### Outside stadia

**Fig. 2.**
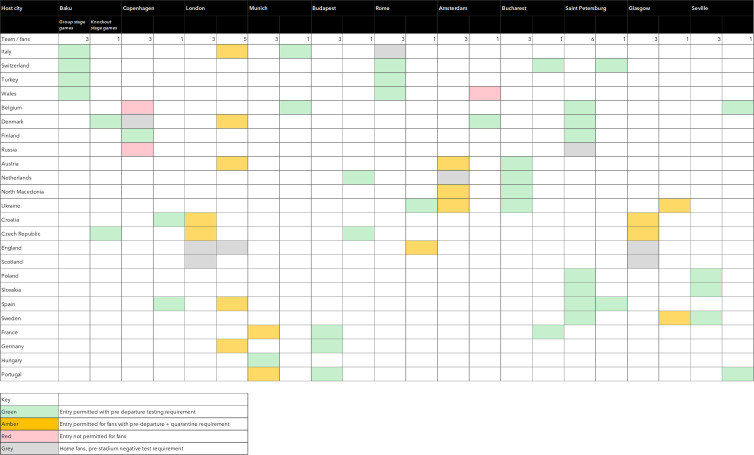
Travel restrictions of international fans during Euro 2020 tournament, 11 June – 11 July 2021.

The ability of away-team fans to travel internationally to attend matches varied across the course of the tournament according to national travel restrictions (Fig. 2). 4/11 host countries offered travel exemptions from mandatory quarantine requirements specifically for fans attending matches: Baku, St. Petersburg, Budapest and Bucharest. The majority of matches (28/51; 54%) allowed for the international travel of away team fans to attend, followed by 17/51 (33%) which had a quarantine and testing requirement and 3/51 (6%) matches which did not allow for international travel of away fans to attend.

Many host countries organised official ‘fan zones’ and ‘football villages’ for fans to view matches on large screens [12]. Although not well documented, there were varied levels of known requirements for fan zones. For example, in numerous fan zones in London, fans were required to register and provide a negative COVID-19 test or proof of vaccination in order to enter fan zones where social distancing was also required. However, other large fan zones such as in Glasgow did not require testing or vaccination.

The increased popularity of hospitality sector venues such as bars and pubs in the wider community showing the games during the tournament represented an additional challenge for mitigation - crowded sports bars are settings where infection prevention and control is difficult. The implementation of PHSM, including measures applied to local businesses such as restaurants and bars, also varied significantly between host and other venues where fans sought to watch the football games outside of their homes (Table 2). The approaches taken here also varied across the European region and across host cities. For instance, in Spain, Italy and Romania, masks were mandatory in all indoor public spaces. In England, fan zones were required by government restrictions to seat groups of fans from the same household at socially distanced tables.

**Table 2.** Public health and social measures outside stadia

Measure	Host requirements
Indoor PHSM	4/11 hosts had mask mandates in place in all indoor public spaces and public transport: Romania, Germany, Russia and Spain. Germany specifically required FFP2 masks in all indoor public spaces and public transport. Italy only required masks in large gatherings. 5/11 hosts only required masks on public transport: Netherlands, England, Scotland, Hungary, Denmark Azerbaijan had no mask mandates in place
Hospitality venues	The majority of hosts had no restrictions on hospitality venues with 9/11 countries operating with all businesses open: Azerbaijan, Romania, Hungary, Denmark, Scotland, England, Germany, Italy, Russia. Netherlands required all fans to present a COVID-19 pass (proof of vaccination or previous infection) at venues. Spain only allowed businesses to open at 75% capacity.
Gatherings	9/11 hosts had capacity restrictions in place on indoor and outdoor gatherings. 3/9 countries (England, Germany and Romania) had strict capacity restriction of under 50 people allowed in indoor gatherings. England – all events limited to 30 people. Germany – indoor events limited to 50 people, outdoor limited to 100. Romania – small group gatherings only. Azerbaijan – public gatherings limited to 150 people. Hungary – events limited to 500 people. Netherlands – all event venues allowed to operate at 60% capacity. Denmark – Indoor events limited to 250 people, outdoor limited to 5000. Scotland – indoor events limited to 100 people, outdoor limited at 1000. Russia – all events limited to 3000. 2/11 hosts had no restrictions on indoor or outdoor gatherings: Spain and Italy

#### Cases identified

Five countries published data on cases, with a total of 9612 cases linked to attendance at Euro 2020 matches. The highest number of linked cases domestically and internationally were reported by England (6376) followed by Scotland (2632), Finland (419), Denmark (170) and Germany (15).

#### Inside stadia

The level of restrictions inside stadia including social distancing, mask mandates, stadium capacity and crowd control were key influences on the risk of transmission at events. Matches with low stadium capacities (below 50%) such as those in Munich (20%), Copenhagen (48%) and Glasgow (23%) generally reported low numbers of cases linked to local matches [13–15]. Munich in particular, which had strict travel restrictions, low stadium capacity and strong enforcement of in-stadia measures only identified five cases linked to attendance.

England reported a stepped increase in the average number of cases per game as stadium capacity increased from 31 during the 3 games at 25% capacity to 247 during 2 games at 50% and 2748 in the final two games at 75%. However, it is important to note that at the final match at Wembley, the 75% stadium capacity limit was unable to be enforced due to mass fan violence and public disorder. The incident, which was later formally investigated as part of independent review found that approximately 2000 ticketless fans forced entry into the stadium [16]

There were clear indications from hosts that adherence to restrictions by fans inside stadia were often low, particularly as the tournament progressed and higher capacities made enforcement challenging for local organisers [13–17]. One post-attendance survey at Wembley suggested as few as 32% of fans wore a mask in the final two games. Some mitigation measures were not enforced by hosts as it was felt it would compromise crowd safety. For example, at the Wembley final pre-entry checks for vaccination and/or testing status were suspended to avoid crowd congestion and potential harm to fans [9–16]

There were a number of factors likely influencing fan behaviour in each of the host cities. As one of the first sporting MG to have taken place since the pandemic began, fans felt a sense of relief from pandemic fatigue, which was prevalent in Europe in the months leading up to the tournament [16–18]. For example in England, the announcement of the upcoming release of all national COVID-19 restrictions, so called ‘freedom day’ also coincided with the tournament. Secondly, for many fans, matches were seen as a once in a lifetime opportunity and were determined to attend. For example, Finnish fans travelled to St. Petersburg to see their national team in a major tournament for the first time or England in the semi-final for the first time since 1996 and a first final [17–19].

#### Travel

Movement of large groups of fans nationally and internationally posed a risk to the transmission of COVID-19. With most host countries allowing away fans entry to attend matches, managing the movement of thousands of fans as well as performing effective COVID-19 checks to reduce transmission risk posed a challenge to local organisers. Finnish authorities faced challenges in managing over 4500 fans crossing the Russian border in groups of coaches and cars after attending a match. The risk of congestion and crowd safety led to over 800 fans being allowed entry without COVID-19 checks, and 419 cases subsequently identified amongst approximately 4500 fans who attended the Finland match in St. Petersburg [19]. Similarly, in Scotland a number of cases were found to be associated with groups of supporters travelling by train, bus or private car and to attend informal gatherings or matches in London.

For ticketholders, all host cities operated a staggered approach to the entry of fans into stadia, with each given a 30-minute slot to enter in order to reduce crowd congestion. However, some organisers reported mixed levels of adherence of fans to time slots [9–17]. For example, London organisers reported low levels of adherence and opted for the final game to allow for the sale of alcohol inside the stadium in an attempt to avoid large crowds of fans entering at once after drinking in nearby pubs [16, 17]

#### Celebrations outside stadia

The global popularity of football and the tournament meant that gatherings and celebrations in public and private spaces to watch matches were common, such as in households, pubs, restaurants and bars. It can be argued that the informal and unstructured nature of gatherings outside stadia in some host countries may have posed a higher risk to the transmission of COVID-19 compared to the more controlled environments in and around stadia. For example, in Scotland and Germany the majority of cases reported (2159/2970; 73%, 123/138; 89%) were associated with attending unofficial Euro 2020 events such as house parties, pubs and restaurant gatherings rather than official matches [15]. Similarly, cases identified in Italy were primarily associated with a ‘frenzy night’ of celebrations across many Italian cities in public squares, private settings and pubs after winning the tournament [20]. Using enhanced surveillance data England also reported a large increase in cases associated with informal gatherings on match days, with spikes of transmission in pubs and bars [15–17]. However, the large number of cases identified in the final match at Wembley highlighted the importance of effective crowd control measures in reducing the risk of infection inside stadia [17].

#### External factors

The COVID-19 pandemic has presented a number of economic, political and scientific challenges for countries to navigate. Sporting MG historically have played an important role in national and international economies in stimulating growth, health and wellbeing. The cancelling of sporting MG has had a large economic impact on the hospitality industry in particular, as well as event organisers and local economies across Europe [21]. The start of the tournament coincided with a phase of the pandemic where restrictions were easing across Europe and countries faced a dilemma of balancing restrictions in order to continue to reduce transmission with the benefits of reopening MG in order to stimulate economic growth. During Euro 2020, host countries navigated the dilemma in different ways according to national decision-making processes and political influences. For example, the decision of stadium capacity during the tournament was based on balancing a number of factors. A recent review found that some hosts felt pressurised by UEFA to achieve a minimum capacity of 25%, with a match previously planned to take place in Dublin relocated as the Irish government would not meet the criteria. Similarly, as the tournament progressed hosts felt pressurised by organisers to increase capacity for ‘showpiece’ matches at the risk of losing hosting if they were unable to meet requirements [16]. Furthermore, increases in stadium capacity at short notice for the Wembley final was felt by local stakeholders to have introduced a degree of uncertainty into planning and made balancing enforcement of COVID-19 measures with crowd safety challenging [16].

#### Surveillance capacity

Robust disease surveillance capacity played a key role in the ability of hosts to detect and respond to the infectious disease threats effectively. A number of countries introduced enhanced surveillance over the tournament. Italy for instance, utilised a combination of event-based and traditional surveillance mechanisms to detect a large increase in cases associated with fan celebrations [20]. Similarly, Germany adopted an enhanced surveillance system taking advantage of strong local public health infrastructures in Munich. Many countries also used contact tracing systems to identify likely sources of infection. Public Health Scotland used a contact tracing surveillance system (‘Test and Protect’) that employed phone interviewers to attach ‘tags’ to cases in order to identify attendance at Euro 2020 matches or informal gatherings [15]. Similarly, the U.K. Health Security Agency used a combination of self-reported surveys and phone interviews of cases by ‘NHS Test & Trace’ to identify potential associations with the tournament [17]. Whole genome sequencing (WGS) was also used in host surveillance systems to identify clusters of cases and likely sources of infection. The Danish Statens Serum Institute utilised a system that combined data from WGS with contact tracing and demographics from a national population-based register [14]. Similarly, Finland took advantage of its high WGS capacity, combining data on cases with contact tracing and demographic information and performing a phylogenetic analysis of the origins of different strains [19].

#### Lessons learnt

The range of experiences of hosting one of the first international MG since the COVID-19 pandemic began has highlighted a number of important lessons for future planning and response. The tournament has shown that MG can happen safely and successfully with robust planning and mitigations in place. Most host countries saw small numbers of cases linked to attendance at matches and found little impact on national rates of COVID-19 infection.

The tournament has also highlighted the often unpredictable nature of football fans and the confluence of social, behavioural and political factors influencing the risk of transmission of infectious diseases. Events at Wembley emphasised the challenge of balancing crowd control and enforcing public health and social measures. It can be argued that the large number of cases picked up in the UK can be attributed to a ‘perfect storm’ of factors that are unlikely to be replicated, such as the timing of the tournament coinciding with the ‘freedom day’ announcement and England reaching a tournament final for the first time in over 50 years. However, future pandemics are inevitable as are the unpredictable nature of football fans. For future MG during complex emergency situations organisers may need to consider a broader range of evidence including recent environmental, social and behavioural studies such as those conducted by the UK Events Research Programme [22]

Strong surveillance capacity in many host countries proved essential in allowing for robust contact tracing and evaluation of the impact of events on COVID-19 transmission. Scotland, England, Denmark and Finland in particular showed strong surveillance abilities to contact, trace and tag attendance at events to cases [14–21]. However, a number of hosts also highlighted the challenges in rapidly tracing contacts and the importance of developing good working relationships with organisers in order to retrieve seating information for example.

Experiences of hosts highlighted the influence and pressure of external factors in balancing public health and social measures. Our understanding of how public health practitioners can manage the broader challenges influencing sporting MG such as economic, social and commercial factors requires significant improvement. Future MG during complex emergencies will require a delicate balance in setting an ‘acceptable’ level of public health risk with the wider impact of cancelling MG on society.

## Data Availability

Data availability is not applicable to this article as no new data were created or analysed in this study.
